# Giant Popliteal Venous Aneurysm—A Rare Cause of Recurrent Pulmonary Embolism

**DOI:** 10.3390/jcm14103548

**Published:** 2025-05-19

**Authors:** Victor Raicea, Oana Mirea, Sebastian Militaru, Mihaela Berceanu, Alexandru Munteanu, Ionuț Donoiu, Liviu Moraru

**Affiliations:** 1 Department of Cardiovascular Surgery, University of Medicine and Pharmacy Craiova, 200349 Craiova, Romania; victor.raicea@umfcv.ro (V.R.); mihaela.berceanu@umfcv.ro (M.B.); 2 Department of Cardiology, University of Medicine and Pharmacy Craiova, 200349 Craiova, Romania; oana.munteanu@umfcv.ro (O.M.); sebastian.militaru@umfcv.ro (S.M.); ionut.donoiu@umfcv.ro (I.D.); 3 Department of General Surgery, University of Medicine and Pharmacy Craiova, 200349 Craiova, Romania; 4 Department of Anatomy and Embryology, George Emil Palade University of Medicine, Pharmacy, Science and Technology of Targu Mures, 540139 Targu-Mures, Romania; liviu.moraru@umfst.ro; 5 Department of Cardio-Vascular Surgery, Emergency Institute of Cardiovascular Diseases and Transplantation, 540136 Targu-Mures, Romania

**Keywords:** popliteal aneurysm, pulmonary embolism, vascular surgery

## Abstract

**Background:** A popliteal vein aneurysm (PVA) is a rare vascular abnormality that can lead to the formation of venous thrombi, resulting in potentially life-threatening pulmonary embolism (PE). **Methods:** We present the case of a 30-year-old female who presented with recurrent pulmonary embolism complicated by cardiorespiratory arrest. Emergency thrombolysis was initiated, which successfully stabilized the patient. Further diagnostic evaluation, including imaging studies, revealed the presence of a giant popliteal vein aneurysm (60/70 mm) as the underlying cause of recurrent embolism. **Results:** The patient underwent surgical repair of the popliteal vein aneurysm to prevent further thromboembolic events. The procedure was performed successfully, and the patient recovered favorably. **Conclusions:** This case underscores the importance of recognizing PVA as a potential cause of recurrent PE, particularly in young patients without typical risk factors.

## 1. Introduction

Popliteal venous aneurysms (PVAs) are rare vascular abnormalities characterized by a localized dilation of the popliteal vein. Studies have shown that PVAs may be an underdiagnosed cause of recurrent or unexplained PE, emphasizing the need for thorough vascular evaluation in patients with embolic events of unknown origin.

## 2. Case Presentation

We present the case of a 30-year-old woman brought to the emergency department after experiencing dizziness an hour prior. On arrival, she was conscious, oriented, with a blood pressure of 120/70 mmHg, a heart rate (HR) of 120 bpm, and an arterial oxygen saturation (SaO_2_) of 97%.

Lung auscultation revealed no murmurs or crackles, while heart auscultation indicated tachycardia without pathological murmurs.

The patient was a former professional tennis player who retired in 2017 and engaged in non-professional training twice weekly. From medical history, we could obtain that the patient underwent left knee ligament surgery in 2016 and experienced an episode of low-risk pulmonary thromboembolism and deep vein thrombosis in 2023, for which she has since been on daily doses of rivaroxaban. However, due to living and receiving medical care abroad, data from previous medical documents, blood tests, or imaging could not be accessed. The rivaroxaban dosage was reportedly halved to 10 mg/day under medical advice two months prior.

EKG showed sinus tachycardia, HR between 125 and 130 bpm, and negative T waves V2-V6.

Focused transthoracic echocardiography demonstrated right ventricular dilation with echocardiographic signs of pressure overload and impaired systolic function with a tricuspid annular plane systolic excursion (TAPSE) of 14 mm. Moderate tricuspid regurgitation was present, with a mildly elevated estimated pulmonary artery systolic pressure (PASP) of 30 mmHg. However, the PASP was likely underestimated given the dense, dagger-shaped appearance of the tricuspid regurgitation jet on continuous wave Doppler. Left ventricular systolic function was normal as calculated by ejection fraction and global longitudinal strain [1]. The inferior vena cava was slightly dilated, with collapse < 50%.

Blood tests showed normal NTproBNP (85 pg/mL) and slightly higher hsTnI of 100 pg/mL. D-dimers were positive, and no other abnormal values were identified.

**Figure 1:**
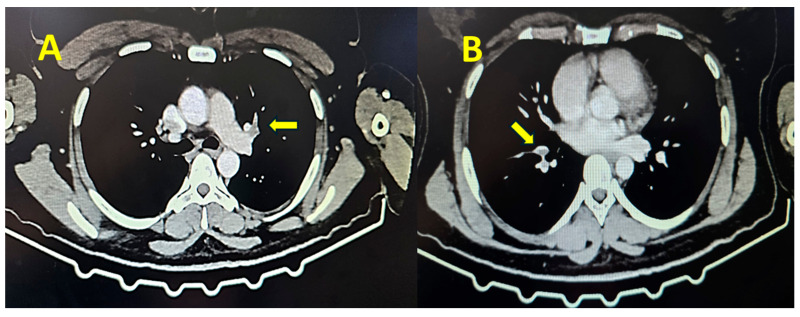
Contrast thoracic computed tomography showing the presence of thrombi at the bifurcation of the left pulmonary artery (**A**, yellow arrow), right superior lobar artery, and segmental arteries (**B**, yellow arrow).

Contrast thoracic computed tomography showed the presence of thrombi in the pulmonary artery, establishing the diagnosis of pulmonary embolism (Figure 1).

The patient was admitted to the cardiology department, and unfractionated heparin was administered first in a bolus of 5000 UI, followed by continuous infusion at 2.5 mL/h. The patient remained asymptomatic, with a systolic blood pressure of 110–120 mmHg, normal oxygen saturation, and sinus tachycardia. Four hours after admission, the patient presented with an abrupt respiratory arrest (SaO_2_ of 60%) followed by a cardiac arrest (asystole on the monitor). Resuscitation maneuvers were immediately started. The patient was ventilated on a balloon with supplemental oxygen, and external cardiac massage was performed with intermittent administration of adrenaline. After 3 min of cardiac resuscitation, the patient recovered consciousness, and a systolic blood pressure of 60 mmHg was obtained. The decision to administer rescue thrombolysis was taken, and Actilyse was administered according to the protocol with clinically favorable evolution (increase in blood pressure and oxygen saturation).

**Figure 2:**
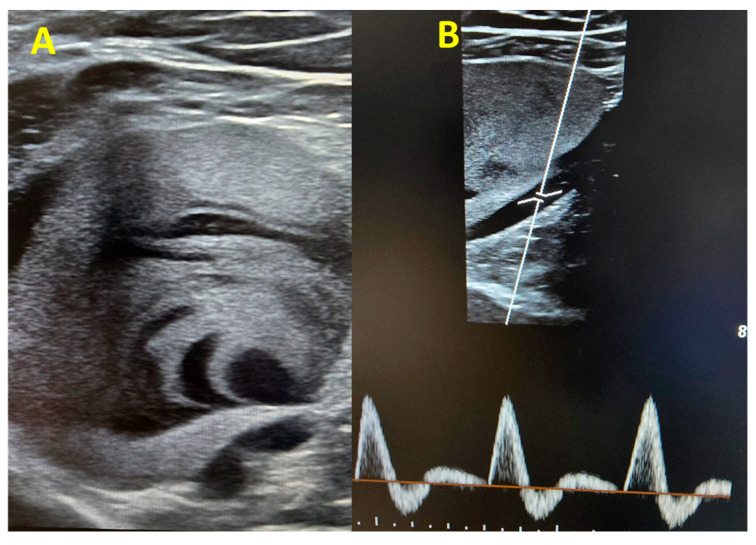
(**A**) Two-dimensional image of the popliteal vein aneurysm. Note the presence of the contrast and the partial thrombosis. (**B**) Normal Doppler PW flux in the popliteal artery.

Within the following hours, a more comprehensive venous Doppler was performed, and we identified a large venous aneurysm in the upper left popliteal area (60/57 mm) with intracavitary thrombosis and intense contrast (Figure 2A). The popliteal artery showed normal caliber triphasic flux (Figure 2B), excluding the possibility of a popliteal artery aneurysm.

**Figure 3:**
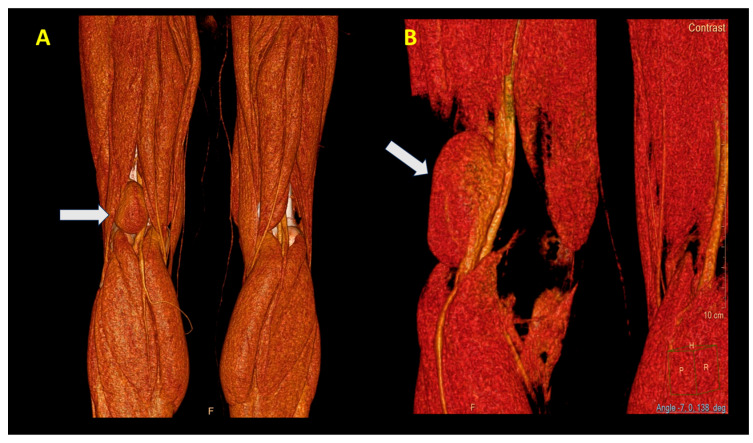
View of the PVA (white arrow). (**A**) Posterior and (**B**) lateral view. Note the large dimensions (60 mm).

An angio CT was performed, which demonstrated the presence of an aneurysm with a communication between the popliteal vein and the cavity (Figure 3A,B).

The patient remained stable, and upon multidisciplinary discussion, the decision was made to undergo surgical correction of the aneurysm.

**Figure 4:**
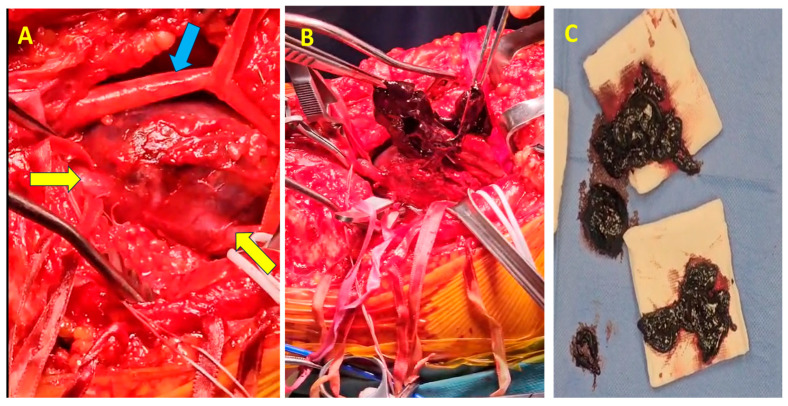
Intraoperative view of the PVA. (**A**) Exposure of the PVA (yellow arrows show the superior and inferior ends of the popliteal vein; blue arrow shows the popliteal nerve). (**B**,**C**) A large amount of thrombus extracted from the aneurysm.

The surgical procedure was conducted with the patient positioned in the ventral decubitus position. Given the substantial size of the popliteal vein aneurysm (60 mm, Figure 4A), circumferential dissection was deemed unfeasible, necessitating a posterior-wall surgical approach (Figure 4B). The vascular control was achieved by clamping both the proximal and distal ends of the aneurysm. Additionally, to reduce venous inflow from the distal segment, the popliteal artery was clamped.

Upon exploration, complete thrombosis of the aneurysm was identified, with a significant volume of semi-fresh thrombus present (Figure 4B,C), which was entirely evacuated. The patency of the proximal and distal segments of the popliteal vein was verified through temporary sequential declamping. A longitudinal incision was made along the entire length of the aneurysm through the posterosuperior wall via an intra-aneurysmal approach. Resection of the entire aneurysm was performed, leaving approximately 2 cm medial and lateral from the normal caliber proximal and distal vein. Reconstruction was achieved by longitudinally suturing the two edges adjacent to the unaffected posterior wall, thereby restoring continuity while preserving the integrity of the non-dilated segment of the popliteal vein.

To prevent the formation of recesses at the junction between the aneurysm and the unaffected segments of the popliteal vein, which exhibited normal proximal and distal calibers, the suturing process was initiated from both extremities adjacent to the unaffected vessel segments.

**Figure 5:**
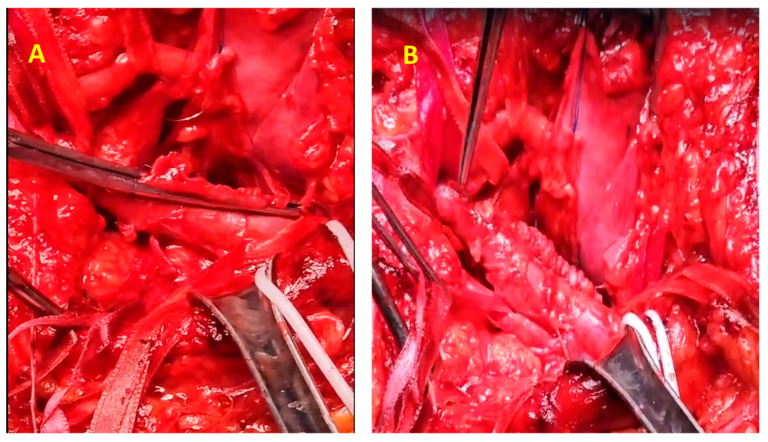
(**A**) Longitudinal suturing of the two edges. (**B**) Final reconstruction of the popliteal vein.

The suture was then completed at the midpoint of the reconstructed area. This approach resulted in a reconstructed popliteal vein with a larger diameter compared to the normal proximal and distal segments of the vessel. Notably, the proximal and distal edges of the normal popliteal vein were asymmetric (13 mm distally and 10 mm proximally), which required adjusted calibration. Given this discrepancy in caliber, a tangential reconstruction was performed. This was achieved by applying lateral clamping to the reconstructed popliteal vein using a straight vascular clamp, precisely matching the dimensions of the unaffected proximal and distal popliteal vein segments (Figure 5A). Recalibration was then conducted in two sequential stages. In the first stage, suturing was performed below the level of the vascular clamp to exclude the traumatized region caused by clamping, particularly considering the thrombogenic risk associated with the patient. The second stage of suturing was carried out following the removal of the vascular clamp, ensuring an optimized reconstruction (Figure 5B).

The postoperative evolution was favorable; the popliteal drainage was suppressed the next day, and the patient was discharged 72 h postoperatively with antiplatelet treatment, oral anticoagulant, and compression stockings for 3 months. The ultrasound control at one week and one month postoperatively showed the patency of the popliteal vein.

## 3. Discussion

Popliteal venous aneurysms (PVAs) are uncommon vascular abnormalities with an incidence of 0.1–0.2% observed during duplex ultrasound examinations [2,3]. Although rare, PVAs represent the second most common location of vein aneurysm after the portal vein [4]. They show a slight left-sided predominance and are more commonly identified in females. Up to 88% PVAs are saccular, and the remainder are fusiform [2,5].

Diagnosis can be challenging due to subtle or nonspecific symptoms. The proposed cut-off to diagnose PVA is 20 mm [6], corresponding to the doubled diameter of a normal popliteal vein, measured in the upright position [7], and larger dimensions are associated with higher risk for embolism.

This case highlights the challenges in diagnosing and managing a large popliteal vein aneurysm in a young, previously healthy patient who presented with recurrent thromboembolic events complicated with cardiorespiratory arrest, which ultimately required urgent surgical intervention. In our case, the aneurysm was of extreme dimensions (up to 60 mm) and extensively thrombosed, explaining the complicated outcome of the second pulmonary embolism.

Although no prior medical records documented the presence of a PVA, we consider that it likely remained undiagnosed and may have been the underlying cause of the previous pulmonary embolic event.

The pathogenesis of PVAs remains incompletely understood, but proposed mechanisms include congenital venous wall weakness, chronic venous hypertension, and trauma. In this case, the patient’s history as a professional sports player, which might have caused repeated venous and muscular traumas, along with knee ligament surgery in 2016, may have contributed to venous abnormalities, although a direct causal relationship is uncertain.

Anticoagulation therapy is often utilized to manage thrombotic risks in patients with symptomatic PVA, and cases demonstrating favorable clinical outcomes have been documented in the literature [7,8]. However, studies have shown that anticoagulation alone does not effectively prevent PE in many patients with symptomatic PVA, as the risk of recurrent embolic events remains high [2,9,10].

Due to the limitations of anticoagulation therapy, additional treatment modalities, such as surgical intervention, are often considered necessary to reduce the risk of recurrent embolization. There is no standardized consensus on the optimal management of PVAs; however, large (>20 mm) or symptomatic aneurysms generally warrant surgical repair to prevent life-threatening thromboembolic complications [5,11,12].

The patient was initially admitted in a hemodynamically stable condition, with no immediate signs of cardiovascular collapse. However, cardiac arrest occurred several hours after admission, highlighting the potential for thromboembolic disease to cause rapid clinical deterioration and raising the possibility that secondary thrombus migration may have taken place following the emergency department CT examination. Such progression is consistent with reports describing delayed hemodynamic compromise in patients with acute PE, particularly when large or mobile thrombi are involved [13,14].

Given that the patient required rescue thrombolysis, surgical treatment was delayed for 9 days after thrombolysis in order to reduce the bleeding risk. Moreover, due to the risk of thrombosis and embolism during this period, the patient was therapeutically anticoagulated with enoxaparin. Although the last dose was administered less than 12 h after surgery, on the morning of the surgery, the venous aneurysm was completely thrombosed, and the risk of embolic recurrence was avoided due to careful surgical manipulation.

Given the substantial size of the aneurysm, circumferential dissection was not feasible, necessitating a posterior-wall approach for direct access. The primary objective of surgical repair was to evacuate the thrombus and restore venous continuity while ensuring proper calibration of the reconstructed segment. The staged tangential reconstruction with lateral clamping was critical in achieving a functional vessel lumen without excessive caliber discrepancies, thereby reducing the risk of post-surgical venous stasis or recurrent thrombosis. The large size of the aneurysm required special attention during the surgery due to its close proximity to the posterior tibial nerve, in order to prevent intraprocedural damage.

The patient had an uneventful postoperative recovery, with serial ultrasound evaluations confirming the continued patency of the popliteal vein. She was discharged with long-term anticoagulation and compression therapy to reduce future thrombotic risks. Given the high recurrence rate of thromboembolism in patients with venous aneurysms, lifelong follow-up with duplex ultrasound is essential for detecting potential complications such as thrombosis, stenosis, or aneurysm recurrence.

## 4. Conclusions

Popliteal venous aneurysm is a recognized but often overlooked cause of pulmonary embolism. Despite its rarity, clinicians should maintain a high index of suspicion, especially in cases of unexplained or recurrent PE without a clear source.

## Data Availability

The original contributions presented in this study are included in the article. Further inquiries can be directed to the corresponding author(s).
